# Phylogeographic pattern suggests a general northeastward dispersal in the distribution of *Machilus pauhoi* in South China

**DOI:** 10.1371/journal.pone.0184456

**Published:** 2017-09-08

**Authors:** Qin Zhu, Bo-Yong Liao, Pei Li, Jun-Cheng Li, Xiao-Mei Deng, Xin-Sheng Hu, Xiao-Yang Chen

**Affiliations:** 1 Guangdong Key Laboratory for Innovative Development and Utilization of Forest Plant Germplasm, South China Agricultural University, Guangzhou, China; 2 State Key Laboratory for Conservation and Utilization of Subtropical Agro-Bioresources, College of Forestry and Landscape Architecture, South China Agricultural University, Guangzhou, China; 3 School of Life Science, Jiaying University, Meizhou, China; 4 State Key Laboratory of Biocontrol and Guangdong Provincial Key Laboratory of Plant Resources, School of Life Sciences, Sun Yat-sen University, Guangzhou, China; University of Innsbruck, AUSTRIA

## Abstract

*Machilus pauhoi* Kanehira is an important timber species in China. A provenance trial was recently set up to evaluate the growth performance of trees from different localities, with the aim of designing seed transfer guidelines. Here, we tested twelve nuclear microsatellite markers derived from other species of the Lauraceae family and investigated population genetic structure in *M*. *pauhoi*. Both the number of observed alleles per locus (*N*_*a*_) and the polymorphic information content (PIC) significantly decreased against the latitude, but showed an insignificant decrease against the longitude. Heterozygosity (*H*_*o*_) and gene diversity (*h*) exhibited a weak correlation with geographic location. Private alleles were present in multiple populations, and a moderate level of population genetic differentiation was detected (*G*_*st*_ = 0.1691). The joint pattern of genetic diversity (*N*_*a*_, PIC, *H*_*o*_, and *h*) suggests that general northeastward dispersal led to the current distribution of *M*. *pauhoi*. Significant but weak effects of isolation-by-distance (IBD) occurred, implicating the mountain ranges as the major barrier to gene flow. Both STRUCTURE and hierarchical clustering analyses showed three distinct groups of populations related to the physical connectivity among mountain ranges. A priority in designing genetic conservation should be given to the populations at the southwest side of the species’ distribution. This conservation strategy can also be combined with the pattern of adaptive genetic variation from the provenance trial for comprehensive genetic resource management of native *M*. *pauhoi*.

## Introduction

**Fig 1:**
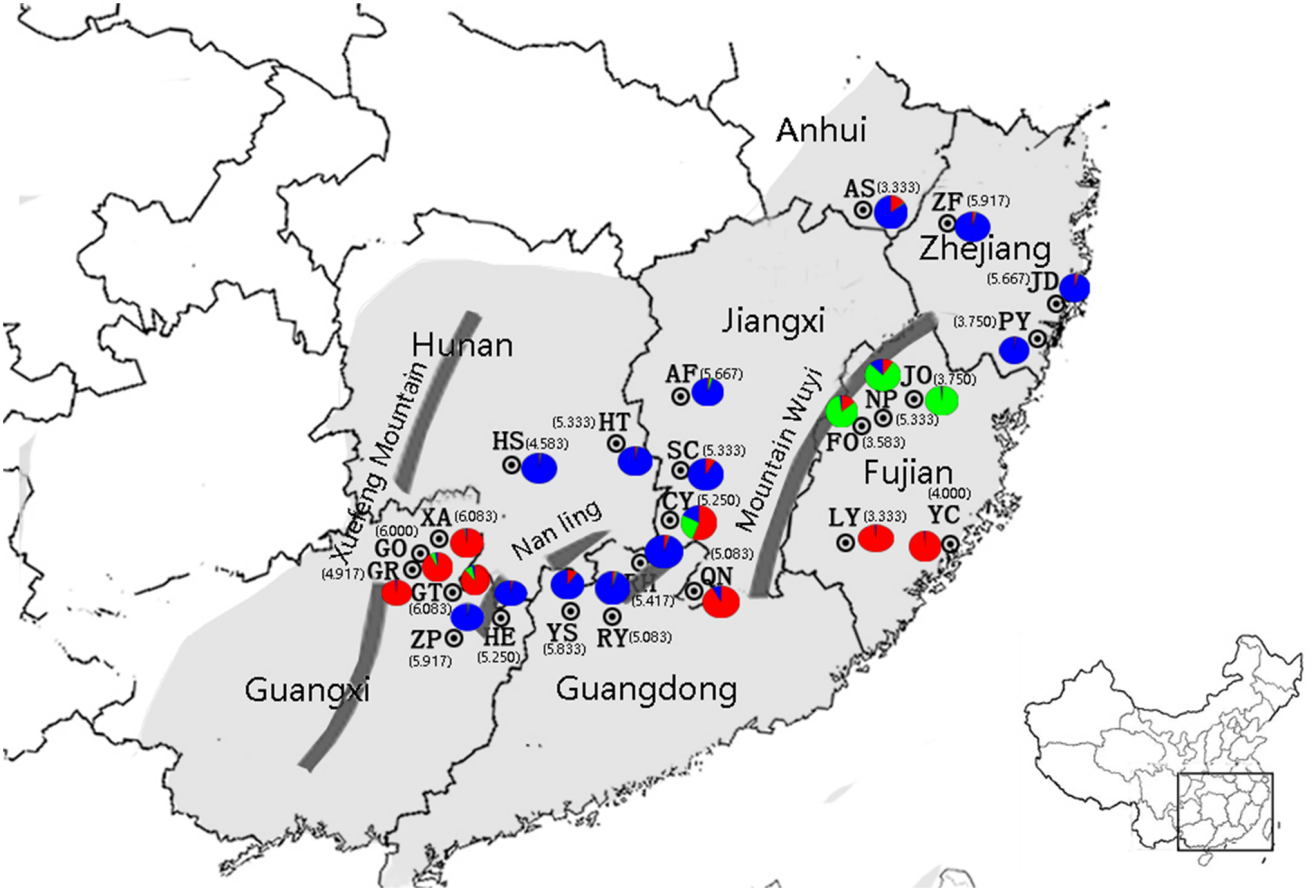
Locations of twenty-four populations in the natural distribution of *M*. *pauhoi*. The natural distribution (in grey) was approximated according to the information on experimental sites from http://www.eflora.c/sp/Machilus%20pauhoi#map and the descriptions of the species’ distribution in [1]. Locality codes are given in Table 1. The numbers beside population codes in parentheses are the numbers of observed alleles per SSR locus (*N*_*a*_). The pie chart at each location represents the proportions of individuals belonging to three groups (in green, blue, and red). The results were derived from the analysis with STRUCTURE at the stationary distributions of all estimates.

*Machilus pauhoi* Kanehira is an important long-lived and evergreen broad-leaved tree species. It is naturally distributed in the subtropical and tropical regions in China, covering Anhui, Zhejiang, Jiangxi, Hunan, Fujian, Guangdong, and Guangxi Provinces (Fig 1) [1]. It often grows in thin thickets on slopes, in the ravine forests below 800m above sea level, or near small streams. In a natural community, the species is often present in a mixed stand with *Castanopsis eyrei* (Champ.) Tutch, *Schima superba* Gardn. et Champ., and *Prunus persica* L. Populations of *M*. *pauhoi* in more distant sites may reside in communities that have independently experienced long-term community successions. The current populations of *M*. *pauhoi* are distributed in patches. Important biological characteristics of this species are that the young seedlings require shade and are adapted to both a fertile acidic soil and an environment of high humidity. However, adult trees show reduced shade requirement and can grow in open sunny habitats [2], [3]. Trees can attain 22m in height and 80cm in diameter at breast height [4]. The species is important for quality timber, besides its conventional use as an ornamental plant or as the raw material for cosmetic perfume and medicine [3], [5], [6].

*M*. *pauhoi* belongs to the Lauraceae family, one of the oldest families of flowering plants. Worldwide, the family includes approximately 50 genera and 2500 to 3000 species [7]. Early fossil records suggest ancient dispersal of the oldest members of the Lauraceae family. These records include dispersal within eastern North America in the mid-Cretaceous [8], within East Asia in the late Cretaceous [9], [10], and within a volcanic Palaeocene oceanic island, the Ninetyeast Ridge in the Indian Ocean [11]. The progenitors of this family expanded to the tropical regions before the Paleogene, and the time for occurrence of this event was earlier than the time of formation of the Himalayan and Hengduan Mountains in Southwest China. Seeds of *M*. *pauhoi* are locally distributed by gravity, rodents, birds, and water transport although the relative contribution of each has not been quantified. This may be similar to the situation in other species of the same family, such as in *Ocotea tenera* [12] and *Neolitsea sericea* (Bl.) Koidz [13]. It is difficult to infer if *M*. *pauchoi* was present in the early Cretaceous period in South China because the speciation time of this species is unknown.

The reproductive system of *M*. *pauhoi* has not been investigated, but other species in the Lauraceae family, including *Ocotea tenera* [12], *Ocotea porosa* [14], and *Neolitsea sericea* [15], exhibit self-compatibility and insect pollination, while *Cryptocarya moschata* Nees (Lauraceae) displays a certain proportion of inbreeding [16]. *M*. *thunbergii* Sieb. Et Zucc exhibits a heterodichogamous system, promoting out crossing [17]. Although no records are available in the literature about the mating system of *M*. *pauhoi*, the species may exhibit a mixed mating system where selfing cannot be excluded because of the structure of perfect flower. Pollination may be mediated mainly by insects, as in *Ocotea porosa* [14], suggesting low inter-population gene flow via pollen dispersal. A field nursery study in Zhanjiang, Guangdong Province, indicated that the seed germination rate was about 80% [18], implying that potential inbreeding depression, if it occurred, was limited. Given these features, we anticipated that population genetic differentiation in *M*. *pauhoi* may be similar to that of other species in the same family (e.g., *Ocotea tenera* [12]), but should be greater than those in wind pollination species or in predominantly outcrossing conifer tree species [19].

The reproductive ecology of *M*. *pauhoi* together with the geological events in the subtropical and tropical regions in China are likely to have determined its pattern of spatial genetic diversity. The formation of the Himalayan (west-east orientation) and Hengduan (southeast-northwest orientation) mountain ranges produce a monsoon climate in South Asia and an arid continental interior in the north subtropical regions [20], [21], [22]. At a large geographic scale, the resultant topography exhibits high altitudes in the southwest areas but low altitudes in the southeast areas in China. A likely common consequence is that many hydrophilous plant species that might previously have occurred in the northern and western regions in the subtropics and tropics, moved to the southeast subtropical and tropical regions where water resource and temperature were not the limiting factors for plant growth. At a small geographic scale, local mountains, such as the Xuefeng, Nanling, and Wuyi mountains in the Southeast China (Fig 1), could reinforce population genetic differentiation by creating barriers to gene flow. Therefore, it is conceivable that population genetic structure in *M*. *pauhoi* has been mainly shaped by local mountain ranges at a small spatial scale.

The purpose of this study is to investigate population genetic variation in *M*. *pauhoi*, and to infer the ecological and evolutionary processes underlying its population genetic structure. An earlier study on population genetic structure in *M*. *pauhoi*, one of three species in Southeast China in the Lauraceae family (*M*. *pauhoi*, *Phoebe chekiangensis*, and *Phoebe bournei*), was confined to two populations at the northeast side of the Wuyi Mountain [23]. The study, using inter-simple sequence repeat (ISSR) markers, showed a lower level of population genetic differentiation (*G*_*st*_ = 0.0702), compared with results in other species of the same family [12]. However, population genetic variation in the range-wide distribution has not been fully examined in *M*. *pauhoi*, and this limits our insights. Here, we investigated the population genetic structure of *M*. *pauhoi* at the range-wide scale.

In our analyses, nuclear microsatellite markers, also known as simple sequence repeats (SSRs), were selected because of many interesting properties of this type of marker, such as the high degree of polymorphism for studying population structure [24] and the potential transferability among species from the same genus or family [25], [26], [27]. Selection of SSR primers from genetically related species is a low-cost, simple, rapid, and effective way to study species possessing low genetic variation. SSR primers have been developed in some other species of the Lauraceae family, such as *M*. *thunbergii*, *M*. *pseudokobu*, and *Persea americana* [28], [29], [30], [31]. Here, we tested the effectiveness of these SSR primers in *M*. *pauhoi*. Through analyzing population genetic structure, we inferred the potential processes responsible for the origin and evolution of the present natural populations of *M*. *pauhoi*. This background information in combination with data on the pattern of adaptive variation derived from a provenance trial can be used to produce a comprehensive genetic resource management plan for *M*. *pauhoi*.

## Materials and methods

### Plant material

Mature fruits were collected from about 16 maternal parents that were located at least 50m apart in each of 24 natural populations in July 2014 (Table 1; Fig 1). Most samples were collected at 100-200m (population codes RH, SC, HT, HE, GR, HS, GT, FO, AS, GO, and NP) or below 100m (YC, JD, PY, ZP, YS, ZF, AF, RY, and JO) above sea level. Three populations were collected at 200-300m (XA, QN, and CY), and one population (LY) at an altitude of 329m above sea level. Annual mean temperature ranges from 16.1°C to 20.5°C (Table 1). Annual rainfall ranges from 1348.7 to 1992.6 mm (1658.22±168.40) across the climatic regions. The climate data were extracted from China Meteorological Data Service Center (CMDC) at http://data.cma.cn/data/index/0b9164954813c573.html, accessed on October 15, 2015. The longitude (N), latitude (E), and altitude data were recorded with hand held GPS equipment (South S760, Guangzhou, China). The fruit samples were washed, and seeds were buried in the wet sand before sowing in soil. Plants were grown in an open experimental site of South China Agricultural University (E113°22’ N23°09’), Guangzhou, China. Annual rainfall is 1689-1888mm, with average relative humidity of 68%, and annual average temperature of 20–22°C according to CMDC records. After a few months, fresh leaves were collected from seedlings of about 40 centimeters in height for DNA extraction.

**Table 1:** Locations, sample sizes, annual rainfall, and annual mean temperature in twenty-four populations of *M*. *pauhoi*.

Populations	Population Code	Longitude(N)/Latitude(E)	Annual rainfall(mm)	Annual mean temperature(°C)	Elevation(m)	Number of samples
Suichuan, Jiangxi	SC	26.33/114.52	1462.0	18.8	104	15
Anfu, Jiangxi	AF	27.38/114.62	1573.1	17.9	81	16
Quannan, Jiangxi	QN	24.75/114.52	1665.0	18.9	249	11
Chongyi, Jiangxi	CY	25.70/114.30	1595.3	18.6	269	6
Jianou, Fujian	JO	27.03/118.32	1673.6	19.1	90	15
Shunchang, Fujian	NP	26.80/117.80	1739.8	18.8	186	15
Quanzhou, Fujian	YC	24.88/118.67	1400.0	19.8	3	15
Jiangle, Fujian	FO	26.73/117.47	1697.0	18.7	146	6
Longyan, Fujian	LY	25.10/117.03	1738.7	20.4	329	4
Xing’an, Guangxi	XA	25.60/110.66	1876.5	18.1	226	16
Lingchuan, Guangxi	GO	25.42/110.33	1926.0	18.7	176	15
Gongcheng, Guangxi	GT	24.85/110.81	1483.8	20.0	142	11
Yangshuo, Guangxi	GR	25.29/110.28	1640.0	19.0	112	8
Hezhou, Guangxi	HE	24.44/111.54	1561.7	20.2	106	12
Zhaoping, Guangxi	ZP	24.18/110.80	1992.6	20.1	52	13
Renhua, Guangdong	RH	25.08/113.75	1660.8	19.9	101	14
Yangshan,Guangdong	YS	24.48/112.63	1845.7	20.5	69	14
Ruyuan, Guangdong	RY	24.35/113.27	1783.8	20.0	86	9
Jande, Zhejiang	ZF	29.48/119.28	1580.5	16.9	74	14
Yueqing, Zhejiang	JD	28.13/120.95	1507.4	17.7	7	15
Pingyang, Zhejiang	PY	27.67/120.57	1785.6	18.3	39	16
Chaling, Hunan	HT	26.79/113.54	1462.0	18.2	105	13
Qiyang, Hunan	HS	26.59/111.85	1348.7	18.5	118	11
Qimen, Anhui	AS	29.87/117.72	1797.7	16.1	150	4

### DNA extraction

About 80mg fresh leaves were used for DNA extraction using an E.Z.N.A. high-performance DNA mini kit (Omega Bio-Tek, Norcross, GA, USA) following the manufacturer’s instructions. The quality of DNA was checked by 1.0% (w/v) agarose gel electrophoresis. The DNA concentration was measured with a Thermo Scientific NanoDrop 1000 spectrophotometer (Thermo Fisher Scientific, Waltham, MA, USA), and adjusted to 50 ng.μL^-1^. Quantified DNA samples were stored at -20°C for PCR amplification.

### SSR amplification and selection of optimum primers

SSR primers were tested according to previous reports on *M*. *thunbergii*, *M*. *pseudokobu*, and *Persea americana* from the Lauraceae family [28], [29]. Twelve pairs of SSR primers were screened to determine the sequence variability observed among 16 plants of *M*. *pauhoi*. These SSR markers are from nuclear genomes rather than organelle genomes, and the resultant amplicons are biparentally inherited. Table 2 lists the SSR primer sequences and the maximum number of observed alleles at each SSR locus.

**Table 2:** SSR primer sequences and the maximum number of observed alleles in *M*. *pauhoi*.

Primer code	Forward primer sequences(5’-3’)	Reverse primer sequences(5’-3’)	Maximum number of alleles
SHRSPa009^†^	ACCCAATCAACAAACAAACCC	CGTTTCCCCAATCCATTTCT	12
SHRSPa010^†^	CGAAGAAGGATAGTCTGAAAACCC	GAGGAAGGATCGGAAGAGAGG	13
SHRSPa012^†^	AACCCTAACGGATTTCAACTAC	ACGGTATAGCTCCTTCCATTC	13
SHRSPa021^†^	ACACCCAACAGATGTGGATAGATAAG	CAGATGAAAAGAACATGGCATTGA	12
SHRSPa057^†^	GCAAGGCATTACGATGTCA	CTCTAGTGGACAAAATCGACAA	7
LMAV03^‡^	CAGAGAATACGGATCTTTGC	GTTCGAAGAAGCCTCAGTTA	11
LMAV15^‡^	TTACCAGTGCTCCTGCTAAT	TGCTCTCAAACCACTTCTCT	28
LMAV24^‡^	CCCTTTCCAAGTTTCCTAAC	GTGCAGAGGTAAGTCACCAT	23
LMAV25^‡^	AGCATGAGAGCAGAATCAAG	ATCCCAACAGAAGCAGTTTA	16
LMAV34^‡^	CCTCAGGTTGAGACCTACTG	CGGTGGCCACTATTAACC	11
LMAV35^‡^	CTCTTCTTTTGCTTTGTTCG	CGTTACGTTAACTGCATCTC	15
ESTAVTC20^‡^	ATTGCTGCATTTTCTTGTTT	AGGGATTAGTCCAACCATTT	20

^†^:Primer codes from [28].

^‡^: primer codes from [29].

PCR amplification was carried out in a 25μL reaction volume that contained 1U *Taq* DNA polymerase (Takara Biotechnology Company Limited, Dalian, China), 1μL 20ng·μL^-1^ template DNA, 2μL 25 mmol·L^-1^MgCl_2_, 0.5μL 10mmol·L^-1^dNTP, 0.5μL10mmol·L^-1^ of each forward and reverse fluorescent primer, and 2.5μL10×PCR buffer (without MgCl_2_, 100 mmol·L^-1^ Tris-HCl, pH 8.8 at 25°C, 500 mmol·L^-1^ KCl). PCR amplification was conducted using the following cycle procedure: an initial 3 minutes of denaturing at 95°C, followed by 10 cycles of three steps (30 seconds of denaturing at 95°C, 30 seconds of annealing at 60°C, and 30 seconds of elongation at 72°C). The next 20 cycles were then conducted with the following steps: 30 seconds of denaturing at 95°C, 30 seconds of annealing at 55°C, and 30 seconds of elongation at 72°C. A final elongation step was set at 72°C for 6 minutes. The PCR products were detected by capillary electrophoresis using an ABI 3730XL DNA analyzer after confirmation of PCR amplification on a 2% agarose gel.

### Data analysis

Population genetic diversity was estimated using POPGENE1.32 [32], including the number of observed alleles (*N*_*a*_) per locus, observed (*H*_*o*_) heterozygosity, gene diversity (*h*), and polymorphic information content (PIC). Gene diversity (*h*) at a locus with *k* alleles was calculated as $1-\sum_{i=1}^{k}{\hat{p}}_{i}^{2}$(*i* = 1,..,*k*) [33]. The PIC at a single SSR locus was calculated as $1-\sum_{i=1}^{k}{\hat{p}}_{i}^{2}-\sum_{i}^{k-1}\sum_{j=i+1}^{k}2{\hat{p}}_{i}^{2}{\hat{p}}_{j}^{2}$ where ${\hat{p}}_{i}$ is the estimate of the frequency of allele *i* (*i* = 1,..,*k*) [34]. GENEPOP version 4.3 [35] was used to test Hardy-Weinberg equilibrium (HWE) at each locus in each population and linkage-disequilibrium (LD) for all pairs of microsatellite loci in each population. Exact tests for HWE were conducted where the p-value in each test was the sum of the probabilities of all possible samples with the same allelic counts [33], [36], [37]. The significant level *α* for testing HWE and LD was adjusted by Bonferroni correction, approximately 0.05/24 = 0.0021 for HWE at each locus and 0.05/(12*11/2) = 0.0008 for LD for each pair of loci. According to the type of repeat motifs flanked by a pair of SSR primers [28], MICRO-CHECKER was applied to checking marker scoring errors due to stuttering, large allele dropout, and null alleles at each locus in each population [38]. Only those loci that were in HWE, absent of null alleles, and in linkage equilibria among them were included for later population genetic analyses. Population genetic differentiation was estimated using Weir and Cockerham’s method [35], denoted by *G*_*st*_ instead of *F*_*st*_ since the number of alleles at a single SSR locus exceeded two alleles among individuals. The significance of *G*_*st*_ from zero was tested through 1000 permutations of the genotype data using GENEPOP[35].

STRUCTURE2.3 [39] was used to further analyze population genetic structure based on the Bayesian method where each individual was assigned to distinct clustering groups with estimated probabilities. The optimum number of clusters (*K*) was identified after 10 independent runs for each *K* value ranging from 1 to 20, with a burn-in of 10,000 iterations followed by 100,000 iterations. We checked that the burn-in of 10,000 iterations was sufficient to ensure the stationary distributions of all estimated parameters, such as *F*_*st*_ and log(alpha) where alpha is the parameter in the prior distribution (Dirichlet) of allele frequencies [39]. *ΔK* was calculated through the second-order changing rate of the likelihood function *L*(*K*) between adjacent *K* values, and the *K* value with a maximum *ΔK* was selected as the appropriate number of clusters [40].

Isolation by distance (IBD) was tested through the regression analysis of *G*_*st*_/(1-*G*_*st*_) on the logarithm of the geographic distance [41]. Note that the geographical distance between two populations was calculated using the longitude and latitude coordinates (Table 1). A Mantel’s test was also conducted to examine the relationship between *G*_*st*_ and the geographic distance [42]. Pearson’s correlations (*r*) were tested between genetic variation (means of *N*_*a*_, *H*_*o*_, *h*, and PIC) and geographic location (longitude, latitude, and elevation). The correlations were also tested between genetic variation and annual rainfall or annual mean temperature.

Nei’s genetic distance was estimated to measure population genetic divergence [43]: *D* = -ln(*I*) where *I* is $I=\frac{\sum_{l}\sum_{u}p_{lu1}p_{lu2}}{(\sum_{l}\sum_{u}p_{lu1}^{2}\sum_{l}\sum_{u}p_{lu2}^{2}{)}^{1/2}}$ in which *p*_*lu1*_ and *p*_*lu2*_ are the frequencies of alleles *u*1 and *u*2 at the *l*th locus from populations 1 and 2, respectively. On the basis of gene frequency data where loci with HWD and null alleles were excluded in their resident populations, we applied pvclust package in R to examining the consensus genetic relationships (1000 bootstrapping samples) among twenty-four populations [44]. A principal coordinate analysis (PCoA) based on the correlation matrix of allele frequencies at multiple loci was conducted to investigate population genetic relationships [45]. PCoA was also conducted on the basis of the correlation matrix using longitude, latitude, elevation, annual rainfall, and annual mean temperature. The two principal coordinate analyses were then compared to view the relationship between comprehensive genetic and ecological components.

## Results

The SSR primers previously reported in *M*. *thunbergii*, *M*. *pseudokobu*, and *Persea americana* were tested. The SSR primers from *M*. *thunbergii* and *M*. *pseudokobu* [30], [31] did not work well, but twelve SSR primers from *Persea americana* [28], [29] were polymorphic. These twelve SSR primer pairs produced a various number of alleles among loci, with a total of 181 alleles ranging from 7 (SHRSPa057) to 28 (LMAV15) (Table 2; S1 Table for genotypes in detail). The alleles from each primer pair and the range for the number of observed alleles from the twelve primer pairs were unequally distributed among populations. There were 37 private alleles in total, and almost all populations had private alleles. For instance, the populations close to the east side of the Xuefeng Mountain had 4, 1, 3, 1, 4, and 2 private alleles in XA, GO, GR, GT, ZP, and HE, respectively. Populations SC, NP, LY, RH, ZF, and PY did not have private alleles. The number of observed alleles per locus (*N*_*a*_) ranged from 3.333± 0.89 (AS) to 6.08± 2.81(XA), with a mean of 5.02±2.20 alleles per locus (Table 3). The *N*_*a*_ values changed from 6.0 in Guangxi Province to 5.0 in Guangdong and Jiangxi Provinces, and to 3.0 or 4.0 in Fujian Province (Fig 1).

**Table 3:** Number of alleles and its range, polymorphic information content (PIC), private alleles, the observed heterozygosity (*H*_*o*_), and gene diversity (*h*) in twenty-four populations of *M*. *pauhoi*.

Population	*N*_*a*_(±Sd)	Range	PIC(±Sd)	Private alleles	*H*_*o*_(±Sd)	*h*(±Sd)
SC	5.33±2.39	(3,10)	0.54±0.19		0.56±0.07	0.59±0.19
AF	5.67±2.64	(2,11)	0.61±0.20	LMAV15(268bp/276bp); LMAV24(204bp)	0.61±0.06	0.65±0.18
QN	5.08±2.27	(1,9)	0.58±0.24	LMAV24(152bp)	0.68±0.08	0.62±0.25
CY	5.25±1.86	(3,9)	0.67±0.11	LMAV35(184bp); ESTAVTC.20(110bp)	0.68±0.05	0.72±0.09
JO	3.75±1.60	(2,7)	0.44±0.23	SHRSPa021(170bp); LMAV15(274bp)	0.44±0.10	0.40±0.256
NP	5.33±1.44	(3,8)	0.56±0.11		0.49±0.07	0.57±0.15
YC	4.00±2.00	(2,8)	0.44±0.21	LMAV03(142bp); LMAV24(158bp/164bp); LMAV34(182bp)	0.49±0.09	0.48±0.23
FO	3.58±1.88	(2,8)	0.46±0.21	LMAV15(244bp/282bp)	0.46±0.10	0.51±0.20
LY	3.33±1.23	(1,5)	0.49±0.22		0.60±0.11	0.55±0.23
XA	6.08±2.81	(3,13)	0.53±0.20	SHRSPa012(144bp); SHRSPa021(196bp); LMAV03(130bp); LMAV15(228bp)	0.59±0.07	0.56±0.20
GO	6.00±2.66	(3,11)	0.59±0.18	SHRSPa057(182bp)	0.58±0.07	0.61±0.21
GT	6.08±2.64	(3,12)	0.59±0.17	SHRSPa021(194bp)	0.64±0.07	0.62±0.16
GR	4.92±1.97	(2,8)	0.53±0.19	SHRSPa012(130bp/140bp); SHRSPa021(176bp)	0.59±0.08	0.54±0.19
HE	5.25±1.71	(3,8)	0.58±0.16	LMAV15(266bp); LMAV24(218bp)	0.67±0.07	0.62±0.16
ZP	5.92±2.23	(3,11)	0.60±0.19	SHRSPa010(126bp/150bp); LMAV03(150bp); LMAV34(154bp)	0.53±0.07	0.63±0.19
RH	5.42±2.19	(2,9)	0.61±0.17		0.64±0.06	0.66±0.16
YS	5.83±2.28	(2,10)	0.60±0.15	SHRSPa021(192bp); LMAV35(212bp)	0.62±0.06	0.65±0.13
RY	5.08±1.83	(3,9)	0.63±0.13	LMAV35(208bp)	0.65±0.06	0.67±0.12
ZF	5.92±2.27	(3,10)	0.59±0.17		0.61±0.07	0.63±0.16
JD	5.67±1.78	(3,8)	0.58±0.19	SHRSPa012(132bp)	0.61±0.08	0.61±0.19
PY	3.75±1.76	(2,6)	0.47±0.24		0.48±0.09	0.48±0.25
HT	5.33±2.19	(3,10)	0.57±0.14	LMAV24(212bp); LMAV35(192bp)	0.58±0.07	0.61±0.14
HS	4.58±2.54	(2,9)	0.49±0.23	LMAV03(164bp)	0.52±0.10	0.53±0.23
AS	3.33±0.89	(2,5)	0.51±0.15	LMAV03(122bp)	0.67±0.09	0.57±0.15
Mean	5.02±2.20		0.55±0.19		0.58±0.02	0.58±0.19

No significant deviations from Hardy-Weinberg equilibrium (HWE) were found in 92.01% (265/288) of the tests in total. Only 7.99% (23/288) of the tests were in Hardy-Weinberg disequilibrium (HWD). The loci with significant HWD were in one to three populations, including SHRSPa009, SHRSPa012, LMAV03, LMAV25, LMAV34, and LMAV35. Loci SHRSPa021, LMAV15, and LMAV24 were in HWD in four, six and five populations, respectively. Population NP had HWD at four loci, while populations SC, CY, FO, LY, HE, RH, RY, JD, HS, and AS had HWE at all twelve loci. All other populations had HWD only at one to three loci.

Tests of linkage disequilibrium (LD) indicated that all SSR locus pairs were in linkage equilibrium in each population (data not shown here). These loci were independent from each other.

The results from MICRO-CHECKER analysis were generally consistent with those of HWE tests (Table 4). Twelve loci with significant HWD were detected to have null alleles, including locus SHRSPa021 in AF, ZP, RH, YS, ZF, and PY, SHRSP057 in HT, LMAV15 in GT, GR, and PY, LMAV24 in QN, and LMAV34 in YC. However, ten loci with significant HWD were detected to be absent of null alleles, including locus SHRSPa021 in GO, LAMV03 in QN, LAMV15 in JO and GO, LAMV24 in XA and GO, LAMV25 in AF, JO, and NP, and LAMV34 in AF. All these loci with significant HWD and null alleles were excluded in their resident populations in further population genetic analyses.

**Table 4:** Tests of Hardy-Weinberg equilibrium at each SSR locus in each population*.

Population	SHRSPa009	SHRSPa010	SHRSPa012	SHRSPa021	SHRSPa057	LMAV03	LMAV15	LMAV24	LMAV25	LMAV34	LMAV35	ESTAVTC20
SC	-0.0476	0.0604	-0.0667	0.5234	-0.2556	0.0526	0.4247	0.1468	-0.1131	0.4677	-0.0509	-0.0051
	(0.1713)	(0.5981)	(1.0000)	(0.0336)	(0.2889)	(0.1141)	(0.0559)	(0.2027)	(0.5445)	(0.0189)	(0.9630)	(0.3603)
AF	0.0991	-0.3084	0.0217	0.4167	0.125	0.4415	0.2500	-0.1194	-0.1498	0.3694	-0.1057	-0.0811
	(0.6046)	(0.3322)	(0.4222)	(0.0023)	(1.0000)	(0.0237)	(0.0055)	(0.2743)	(0.9288)	**(0.0000)**	(0.0040)	(1.0000)
QN	-0.1364	-0.1111	-0.2587	-	-0.3423	0.1954	-0.0588	0.5714	-0.2088	-0.2422	0.3370	-0.0471
	(1.0000)	(0.8337)	(0.6761)		(0.1735)	**(0.0019)**	(0.7272)	**(0.0000)**	(0.1009)	(0.9691)	(0.0025)	(0.3214)
CY	0.5122	0.1429	-0.1364	0.2857	0.2308	0.1304	0.1304	0.1837	0.0741	0.1111	0.2727	-0.0526
	(0.0707)	(0.3016)	(0.6081)	(0.5152)	(0.5688)	(0.5844)	(0.4533)	(0.2249)	(0.6815)	(1.0000)	(0.1918)	(1.0000)
JO	0.1411	0.6500	0.7395	0.3538	-	-0.1493	0.3450	0.0508	-0.6216	-	-0.1957	-0.098
	(0.0127)	(0.1034)	**(0.0003)**	(0.0805)		(1.0000)	**(0.0000)**	(1.0000)	**(0.0010)**		(0.1270)	(0.5540)
NP	0.4024	0.2186	0.5584	0.3935	0.2632	-0.1789	0.6340	0.2065	-0.3333	0.5692	-0.0633	0.3022
	**(0.0000)**	(0.1588)	**(0.0008)**	(0.0157)	(0.0618)	(0.0258)	**(0.0000)**	(0.0284)	**(0.0002)**	(0.0115)	(0.0075)	(0.0563)
YC	-0.3874	-	-0.1667	-0.0500	0.1064	0.3333	0.4118	0.0261	-0.3047	-0.1879	0.6769	-0.3291
	(0.2042)		(1.0000)	(1.0000)	(0.2630)	(0.1332)	(0.0501)	(0.1212)	(0.5431)	(1.0000)	**(0.0003)**	(0.1082)
FO	-0.1429	1	1	0.1489	1	-0.1429	0.1071	0.6429	-0.2000	-0.1111	0.5238	-0.4706
	(0.6364)	(0.0909)	(0.0909)	(0.5290)	(0.0909)	(0.6364)	(0.0963)	(0.0909)	(0.0953)	(0.6306)	(0.0389)	(0.6364)
LY	-0.0909	-	0.0526	-0.1250	0.3684	0.2941	-0.3333	-0.2000	-0.2000	-	1	-0.2000
	(1.0000)		(1.0000)	(1.0000)	(0.3143)	(1.0000)	(1.0000)	(0.4667)	(1.0000)		(0.0857)	(1.0000)
XA	0.0270	-0.1180	-0.1290	0.3531	0.1892	0.1388	0.2643	-0.1509	-0.2963	-0.1194	-0.0942	-0.0448
	(0.5649)	(1.0000)	(1.0000)	(0.0606)	(0.3088)	(0.1914)	(0.0236)	**(0.0000)**	(0.2039)	(1.0000)	(0.1559)	(0.8606)
GO	-0.1290	0.1040	-0.0678	0.5440	0.0311	-0.1507	0.1111	0.2861	-0.0490	0.1340	0.1366	-0.1475
	(1.0000)	(0.1418)	(0.1980)	**(0.0015)**	(0.1367)	(1.0000)	**(0.0006)**	**(0.0000)**	(0.2263)	(0.7061)	(0.0331)	(0.3813)
GT	0.3043	0.4828	0.2381	-0.2579	-0.3793	-0.0909	0.4872	0.1351	-0.1579	0.0062	-0.2403	0
	(0.0299)	(0.0146)	(0.2204)	(0.2374)	(0.3560)	(0.5046)	**(0.0000)**	(0.0273)	(0.6648)	(0.9041)	(1.0000)	(0.9039)
GR	0.0345	-0.1053	0.1765	-0.3067	-0.5806	-0.0769	0.6000	0	-0.1807	0.4085	0.1600	-0.1667
	(0.5897)	(1.0000)	(0.3849)	(0.6738)	(0.1385)	(1.0000)	**(0.0002)**	(0.0585)	(0.6308)	(0.0385)	(0.3846)	(0.9336)
HE	-0.0233	-0.0820	-0.0405	0.1098	-0.0051	0.2222	0.2617	-0.1524	-0.2279	0.0365	-0.2166	-0.0645
	(0.6183)	(0.7966)	(0.4939)	(0.6067)	(0.5205)	(0.2443)	(0.0389)	(0.5923)	(0.7920)	(0.7416)	(0.6370)	(1.0000)
ZP	0.1795	0.2441	-0.1368	0.7798	0.1845	0.0943	0.4504	0.0649	-0.0286	0.4545	0.1367	-0.0588
	(0.0744)	(0.1186)	(0.3535)	**(0.0000)**	(0.6946)	(0.2790)	(0.0162)	(0.2516)	(0.8156)	(0.0209)	(0.7458)	(1.0000)
RH	0	-0.0554	-0.1818	0.5612	-0.1440	0.0076	0.3839	-0.0130	0.0158	-0.1038	0.1280	0.1415
	(1.0000)	(0.8883)	(1.0000)	(0.0169)	(0.7321)	(0.0545)	(0.0068)	(0.1298)	(0.2391)	(0.5375)	(0.6770)	(0.5395)
YS	-0.0970	-0.0648	-0.2053	0.7301	0.0449	0.2121	0.2255	-0.0947	-0.2423	0.2857	0.1503	0.0667
	(0.3076)	(0.9309)	(1.0000)	**(0.0000)**	(0.1179)	(0.3406)	(0.1882)	(0.0376)	(0.6494)	(0.5704)	(0.2015)	(0.1491)
RY	-0.0182	-0.0667	0.2941	0.2889	0.3600	0.1919	0.2308	-0.152	-0.2000	-0.0256	0.2558	0.0943
	(0.4650)	(0.8431)	(0.0588)	(0.0949)	(0.0694)	(0.7016)	(0.2009)	(0.6620)	(1.0000)	(1.0000)	(0.0922)	(0.3696)
ZF	0	0.0983	-0.0400	0.6953	-0.1005	-0.0493	0.2340	-0.1629	-0.1223	-0.0331	-0.0288	0.3839
	(1.0000)	(0.5406)	(1.0000)	**(0.0004)**	(1.0000)	(0.3480)	(0.0326)	(0.0229)	(0.8999)	(0.6290)	(0.7003)	(0.0304)
JD	-0.1168	-0.0301	-0.1407	0.3957	-0.1375	0.3194	0.2548	-0.1362	-0.1064	-0.0667	-0.0312	0.5692
	(0.3545)	(0.7358)	(0.8255)	(0.0024)	(0.3816)	(0.1121)	(0.0542)	(0.0132)	(0.5142)	(1.0000)	(0.5651)	(0.0115)
PY	-0.1932	-0.1321	0.0741	0.4721	-0.0345	-0.2500	0.7794	-0.2064	-0.1141	0.4767	-0.068	-
	(0.0844)	(1.0000)	(0.6086)	(0.0030)	(1.0000)	(0.5432)	**(0.0000)**	**(0.0007)**	(0.1373)	(0.1081)	(0.8357)	
HT	0.0182	0.0357	0.0476	0.2453	0.7241	0.0254	0.3909	-0.5522	-0.1852	0.1765	0.2421	0.0828
	(0.3028)	(0.9364)	(0.5230)	(0.2719)	(0.0024)	(0.9181)	(0.0044)	**(0.0000)**	(0.5723)	(0.2220)	(0.0439)	(0.5782)
HS	-0.3223	-0.0204	0.0566	0.6552	0.0566	0.3197	0.6429	-0.3665	0.0816	0.5745	-0.087	0.6552
	(0.6698)	(0.7782)	(1.0000)	(0.0476)	(1.0000)	(0.0295)	(0.1429)	(0.0042)	(0.0317)	(0.1084)	(0.9940)	(0.0476)
AS	0	-0.5	-0.0909	1	-0.2857	-	0.3333	-0.3333	-0.3333	0	-0.2632	-0.2000
	(1.0000)	(0.5429)	(1.0000)	(0.0286)	(1.0000)		(0.3143)	(1.0000)	(1.0000)	(0.3143)	(0.7714)	(1.0000)

*: Estimates of inbreeding coefficients (*F*_*is*_) are shown in the table. P-values for the exact test of HWE are shown in parentheses. The numbers in bold indicate that *F*_*is*_ was significantly different from zero based on the significant level with Bonferroni correction. Symbol”-”means that the results were unavailable from the analysis with GENEPOP [35].

The average PIC was about 0.55±0.19, with the highest value in CY (0.67±0.11) and the lowest value in JO (0.41±0.23). Populations JO, YC, PY, FO, LY, and HS had the PIC values of less than 50%. Mean observed heterozygosity (*H*_o_) in each population was close to the average gene diversity that is equal to the expected heterozygosity (*H*_e_) under HWE (Table 3).

**Fig 2:**
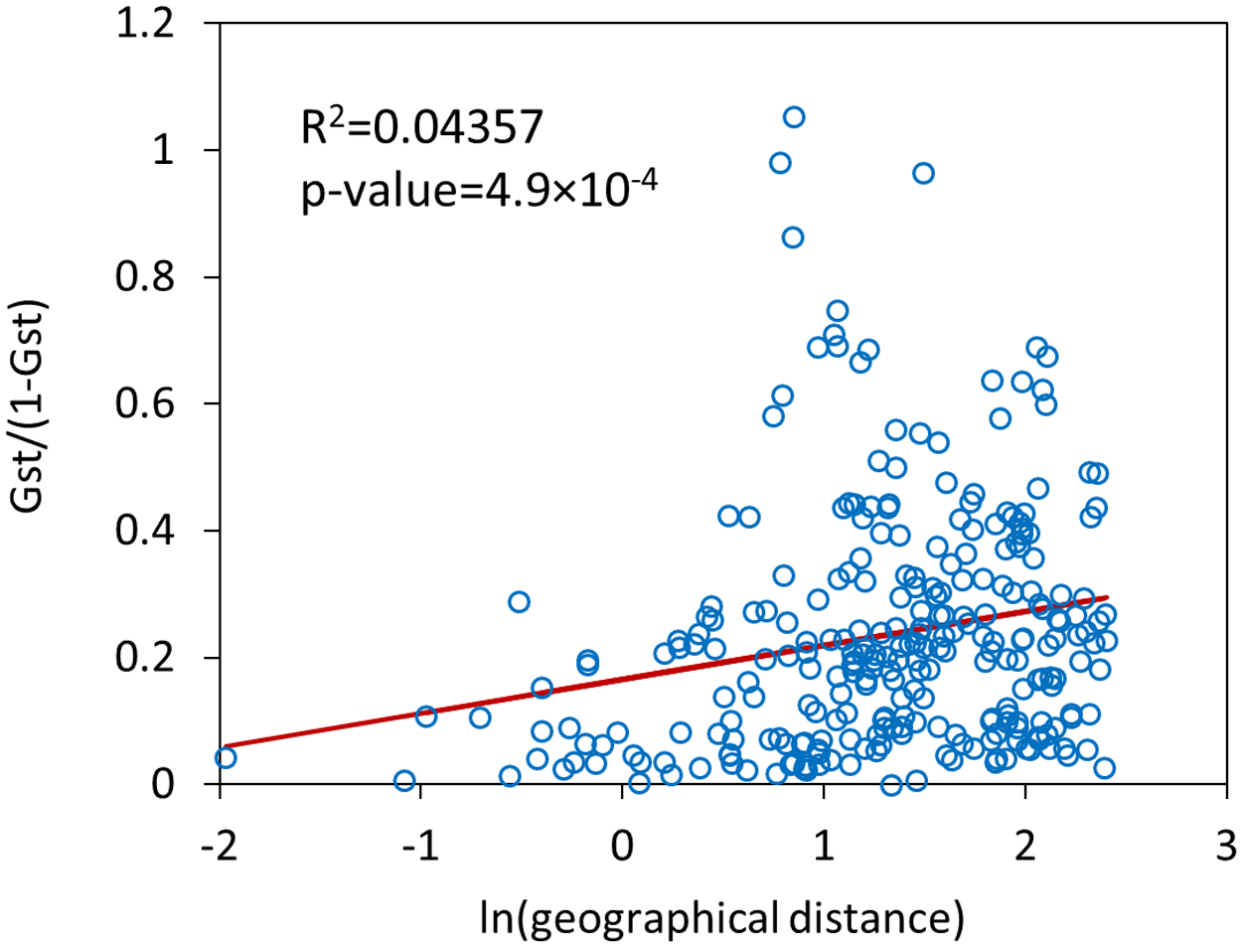
Test of the effects of isolation-by-distance (IBD). IBD effects on population differentiation were significant from the regression analysis: *G*_*st*_/(1-*G*_*st*_) = 0.1657+ 0.0536ln(geographic distance).

Analysis of population genetic differentiation indicated that 16.91% of the total genetic variation at multiple SSR loci was distributed among populations. Genetic differentiation at individual loci was significantly different from zero (p-value<0.001; Table 5), with the *G*_*st*_ values ranging from 0.0850 at the LMAV24 locus to 0.3146 at the SHRSPa010 locus. When the inverse number of migrants between populations inferred from *G*_*st*_/(1-*G*_*st*_) was regressed on the logarithm of geographic distance, a significant relationship occurred at loci SHRSPa009, LMAV03, LMAV25, and LMAV35, but not at the remaining loci (Table 5). A multilocus analysis indicated that a significant but very weak IBD effect occurred (*a* = 0.1657 and *b* = 0.0536; p-value = 0.0005; *R*^2^ = 0.0436; Fig 2). Mantel’s test based on the multilocus *G*_*st*_ and geographic distance matrices also supported the presence of weak IBD effects at multiple loci (the observed correlation *r* = 0.2661, p-value = 0.01).

**Table 5:** Population genetic differentiation and isolation-by-distance tests at individual SSR loci in *M*. pauhoi*.

Locus	*G*_*st*_	*a*	*b*	p-value	*r*
SHRSPa009	0.0976	0.0860(0.0296)	0.0514(0.0195)	0.0091	0.1853
SHRSPa010	0.3146	0.4497(0.0893)	0.1053(0.0595)	0.0778	0.1102
SHRSPa012	0.1015	0.1591(0.0532)	0.0416(0.0360)	0.2498	0.0910
SHRSPa021	0.2307	0.4679(0.1248)	0.0018(0.0849)	0.9827	0.0017
SHRSPa057	0.1978	0.2968(0.0919)	0.0551(0.0609)	0.3663	0.0586
LMAV03	0.1422	0.1431(0.0537)	0.0799(0.0346)	0.0219	0.1563
LMAV15	0.0919	0.1133(0.0332)	0.0361(0.0233)	0.1246	0.1409
LMAV24	0.0850	0.1180(0.0321)	0.0058(0.0208)	0.7802	0.0238
LMAV25	0.0979	0.0886(0.0133)	0.0232(0.0089)	0.0093	0.1754
LMAV34	0.2702	0.6382(0.2158)	0.0286(0.1423)	0.8411	0.0142
LMAV35	0.1238	0.1242(0.0180)	0.0274(0.0120)	0.0228	0.1475
ESTAVTC20	0.2722	0.3591(0.0638)	0.0671(0.0421)	0.1129	0.0997
Multilocus	0.1691	0.1657(0.0228)	0.0536(0.0152)	0.0005	0.2087

*: All multilocus *G*_*st*_ values were significant from zero (p-value<0.001). *a* and *b* are respectively the intercept and regression coefficient in *G*_st_/(1-*G*_st_) = *a*+*b* ln(geographic distance) analysis; *r*: Pearson’s correlation coefficient between *G*_*st*_/(1-*G*_*st*_) and geographic distance. Standard errors for estimates *a* and *b* are given in parentheses.

Pearson’s correlation tests indicated that genetic variation (*N*_*a*_, PIC, except *H*_*e*_ and *H*_*o*_) significantly decreased against latitude, but decreased insignificantly against longitude or elevation. Genetic variation did not significantly correlate with the annual rainfall or the annual mean temperature (Table 6).

**Table 6:** Pearson correlations of the genetic variation (the number of observed alleles per locus, polymorphic information content, observed heterozygosity, and gene diversity) with the geographic location (longitude, latitude, and elevation) and with the climate factors (annual rainfall and annual mean temperature) in *M*. pauhoi*.

Genetic variation	Longitude	Latitude	Elevation	Annual rainfall	Annual mean temperature
*Average N*_*a*_	-0.2601(0.2197)	-0.5303(0.0077)	-0.1132(0.5985)	0.0585(0.7859)	0.0827(0.7009)
*Average PIC*	-0.2366(0.2656)	-0.4235(0.0392)	0.1290(0.5480)	0.1335(0.5338)	0.0860(0.6893)
*Average H*_*o*_	-0.1519(0.4787)	-0.3465(0.0972)	0.3096(0.1410)	0.0020(0.9926)	-0.0271(0.9001)
*Average h*	-0.2173(0.3077)	-0.3597(0.0843)	0.1312(0.5411)	0.0042(0.9843)	0.0814(0.7054)

*: P-values for testing correlations are shown in parentheses.

**Fig 3:**
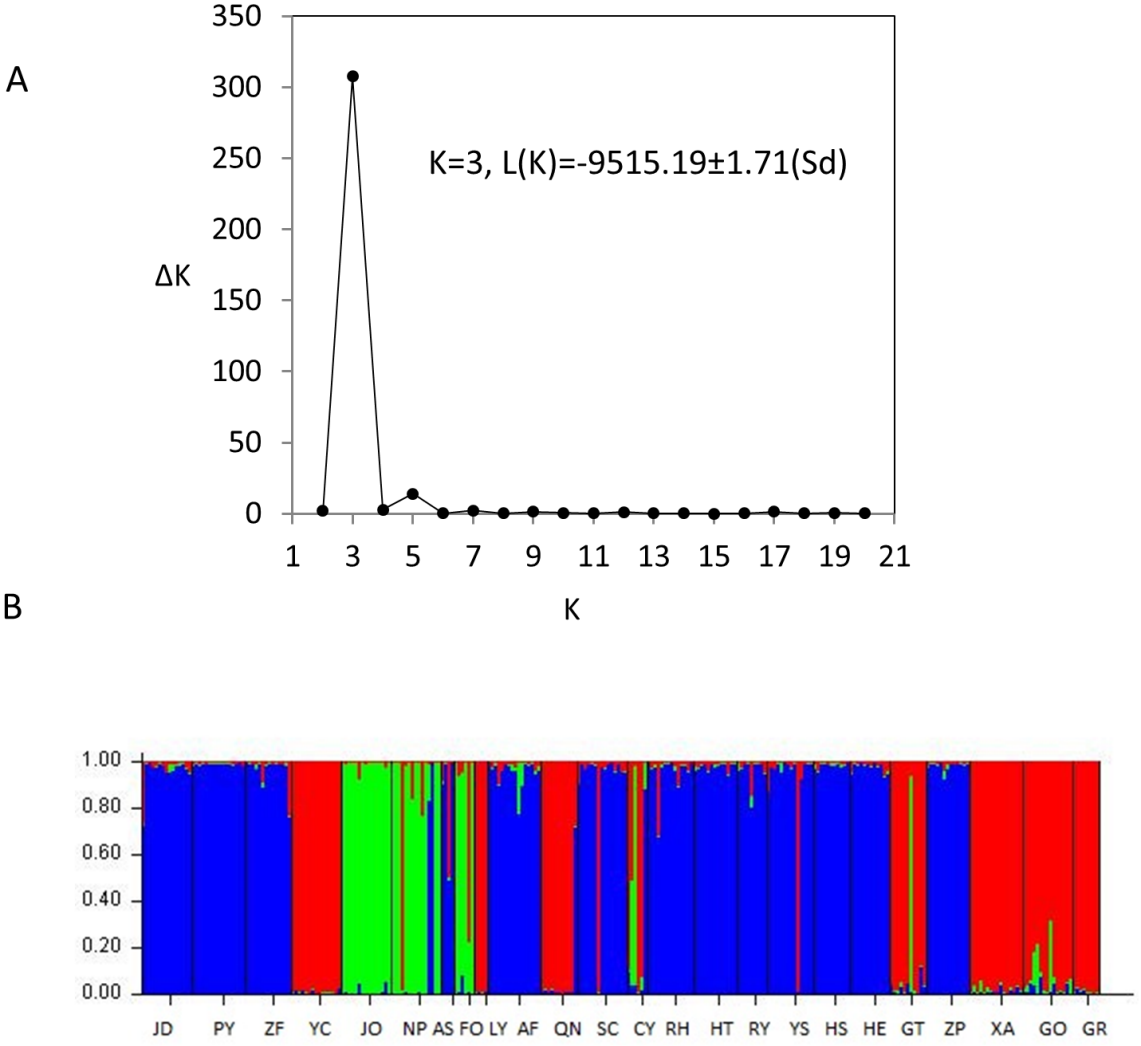
Population genetic structure in *M*. *pauhoi*. A: a relation between the number of determined group *K* and the estimated value *ΔK*; B: a clustering analysis for *K* = 3 with 24 populations.

Analysis with STRUCTURE showed a maximum *ΔK* value at *K* = 3(*ΔK* = 307.66), indicating that the twenty-four populations were appropriately divided into three groups (Fig 3). Group I (in green) included JO, NP, and FO in Fujian Province. Group II (in red) included GO, GT, GR, and XA from Guangxi Province, YC and LY from Fujian Province, and CY and QN from Jiangxi Province. Group III (in blue) included SC and AF from Jiangxi Province, HE and ZP from Guangxi Province, ZF, PY, and JD from Zhejiang Province, YS, RY, and RH from Guangdong Province, HT and HS from Hunan Province, and AS from Anhui Province. When three groups of populations were mapped to the geographic locations (Fig 1), Group II was separated into two parts, with four populations (GO, GT, GR, and XA) being close to the Xuefeng Mountain and the other four populations (YC, LY, QN, and CY) at the east sides of the distribution. Populations in Group III covered a wide range of distribution between the Xuefeng and Wuyi Mountains.

A further finer scale analysis indicated that populations in Group I exhibited small but significant genetic differentiation (multilocus *G*_*st*_ = 0.004, p-value = 0.0031). Population JO was significantly divergent from NP (*G*_*st*_ = 0.0141, p-value = 0.0142) and FO (*G*_*st*_ = 0.0591, p-value = 0.0293). However, population NP was not significantly divergent from FO (*G*_*st*_ = 0.0060, p-value = 0.2340). Populations in Group II exhibited highly significant differentiation (*G*_*st*_ = 0.1111, p-value<0.0000). Each pair of populations exhibited significant divergence at each locus. Populations in Group III also exhibited highly significant differentiation (*G*_*st*_ = 0.0748, p-value<0.0000). All pairwise populations exhibited significant differentiation except between ZF and JD (*G*_*st*_ = 0.0174, p-value = 0.1262). Population differentiation between Groups I and II ranged from *G*_*st*_ = 0.0956 (NP-CY) to 0.4954 (JO-YC), with the mean of 0.3138±0.1008. This was slightly different from population differentiation between Groups I and III that ranged from *G*_*st*_ = 0.1944 (NP-RY) to 0.5129 (JO-PY), with the mean of 0.3129±0.0707. Population differentiation between Groups II and III was relatively small and ranged from 0.0458 (CY-RY) to 0.4072 (YC-PY), with the mean of 0.1851±0.0632.

**Fig 4:**
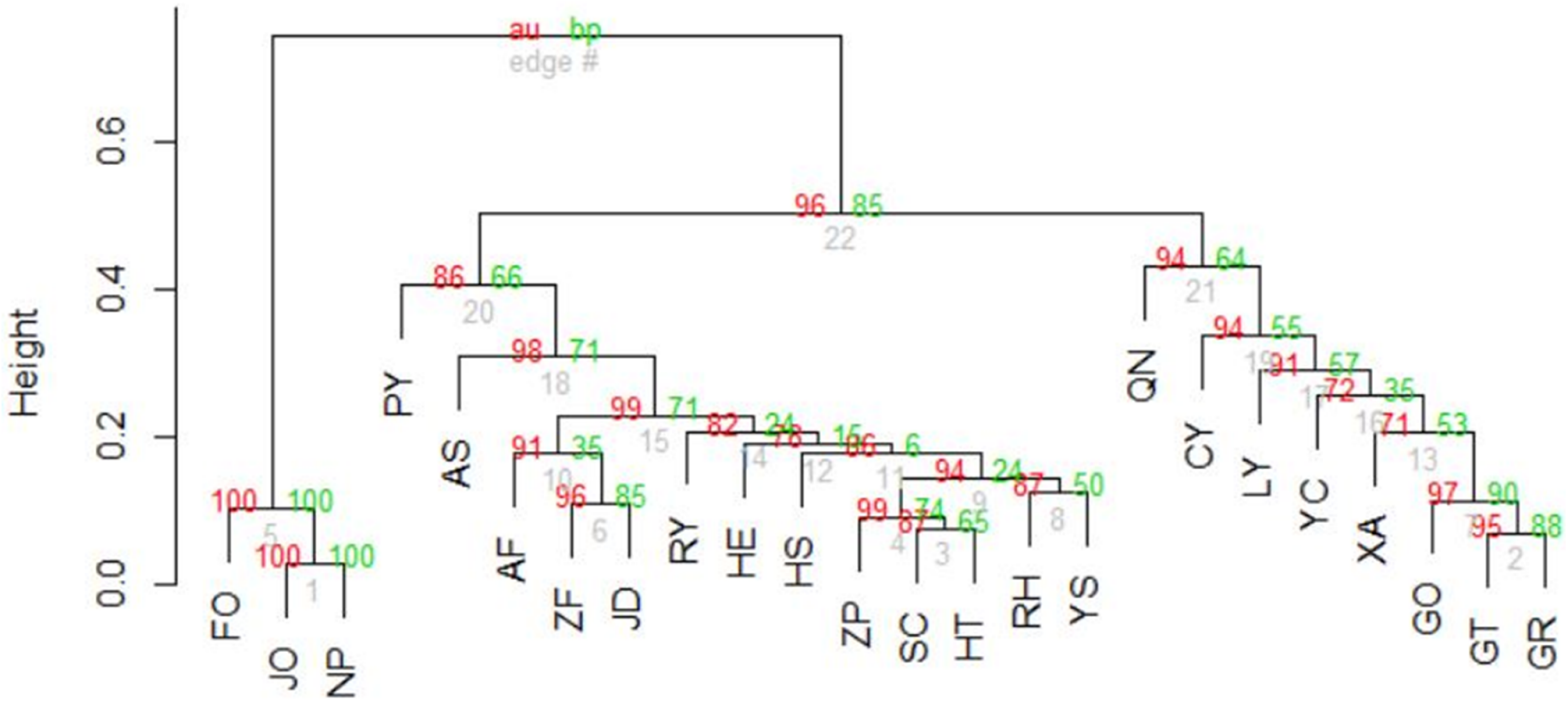
A consensus tree of genetic relationships among twenty-four populations in *M*. *pauhoi*. Pvclust package in R was used to cluster populations according to the correlation matrix among gene frequencies at multiple loci [44]. The numbers at the forks were the percentages of approximately unbiased (AU; in red) p-values and bootstrap probabilities (BP, in green) estimated from 1000 bootstrapping samples.

Nei’s genetic distances (*D*) between populations ranged from 0.0278 (JO-NP) to 2.4169 (JO-QN), with a mean of 0.5389±0.3590. A consensus tree of genetic relationships showed three distinct groups among 24 populations (Fig 4), the same as those derived from the analysis with STRUCTURE (Fig 3). Populations in Group I (JO, NP, and FO) were closely related but separated from the other populations with a high probability (100/100). Nei’s genetic distances between populations in Group I ranged from 0.0278 (JO-NP) to 0.1018 (JO-FO), with a mean of 0.0718±0.0390. Populations in Group II were joined with a probability of 0.64 (bootstrap probability (BP) p-value) or 0.94 (an approximately unbiased (AU) p-value). However, the four populations from Guangxi Province (GO, GT, GR, and XA) were separated from LY and YC in Fujian Province with an intermediate probability (0.35 for BP p-value and 0.71 for AU p-value). Nei’s genetic distances between populations in Group II ranged from 0.0599(GT-GR) to 0.5252 (GT-QN), with the mean of 0.2934±0.1190. Group III was closely related to Group II but distinct from Group I. Nei’s genetic distances between populations in Group III ranged from 0.0650(SC-HT) to 0.6896 (AS-PY), with the mean of 0.2305±0.1132. Altogether, Group I had the closest genetic relationship among populations (JO, NP, and FO), followed by the increased genetic divergence on average among populations in Group III and Group II.

**Fig 5:**
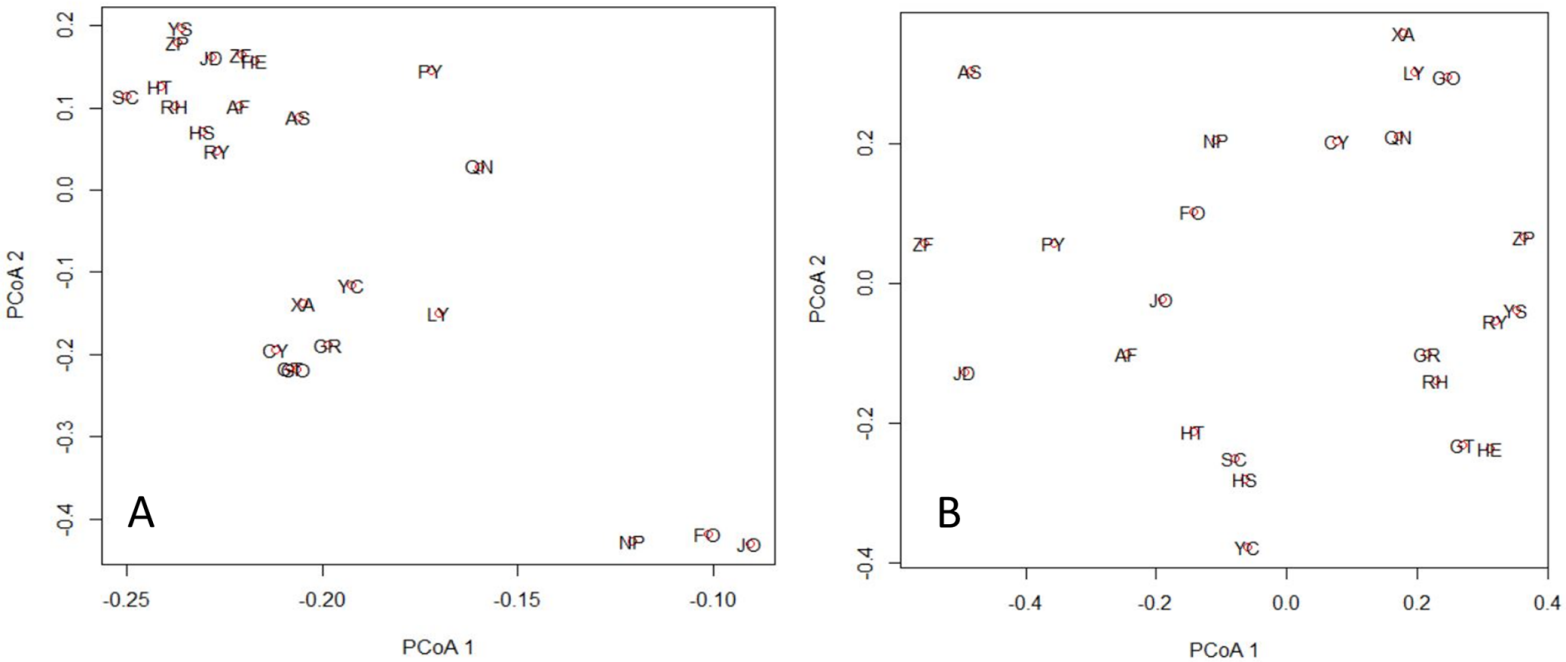
Principal coordinates analysis (PCoA) of twenty-four populations in *M*. *pauhoi*. A. PCoA was based on the correlation matrix of allele frequencies at 12 loci. The first and second principal components accounted for 57.96% and 12.67% of genetic variation, respectively. B. PCoA was based on the five ecological factors (Table 1). The first and second principal components accounted for 26.32% and 14.67% of variation, respectively.

Analysis of principal coordinates supported the three distinct groups among the twenty-four populations (Fig 5A). The first two principal components jointly accounted for 70.63% of total genetic variation. Three populations in Group I (JO, FO, and NP) were clearly separated from the remaining 21 populations. Groups II and III could also be separated although they were more closely related, consist with the results in Figs 3B and 4. However, the three groups could not be delineated from the map of the first two principal components of ecological factors (jointly accounting for 41% of variation; Fig 5B), indicating discordant relationships between comprehensive genetic and ecological components.

## Discussion

Analysis of genetic diversity at twelve nuclear SSR loci indicated that the number of observed alleles per locus (*N*_*a*_) and genetic diversity (PIC) significantly decreased along the southwest-to-northeast mountain ranges, but the average heterozygosity (*H*_*o*_) and gene diversity (*h*) did not. These results imply that a general northeastward dispersal of *M*. *pauhoi* led to its present distribution in South China. Private alleles were present in multiple populations, and about 16.91% of genetic variation was distributed among populations. Both population structure and clustering analyses supported three groups of populations in the natural distribution of *M*. *pauhoi*. Significant but weak effects of isolation-by-distance existed, suggesting that the barrier to gene flow mainly arose from physical mountains.

The transferability of nuclear SSR markers may vary with species or families owing to the high mutation rate at SSR loci in eukaryote organisms. Although primer sequences are often not conserved even among genetically related species due to the high mutation rate, preliminarily screening of microsatellite primers from within the same genus or family may nevertheless yield useful marker loci. Here, we tested the effectiveness of interspecific transferability in *M*. *pauhoi* with the SSR markers previously derived from *M*. *thunbergii*, *M*. *pseudokobu*, and *Persea americana*. We observed that the SSR primers previously explored for *M*. *thunbergii* and *M*. *pseudokobu* [30], [31], did not work well in *M*. *pauhoi*, but some SSR primers from *Persea americana* worked well. Further, the absence of LD among loci implied that these loci were freely recombined if they were on the same chromosomes or on different chromosomes. Our results indicated that the SSR markers employed were available for investigating genetic variation in *M*. *pauhoi*.

The genetic diversity or heterozygosity in natural populations at the SSR loci that were different from those we examined was quite variable in *Lindera glauca* [46], *Cinnamomum camphora* [47], *Ocotea species* [48], *Aniba rosaeodora* [49], and *Beilschmiedia roxburghiana* [50]. As expected, the heterozygosity at microsatellite loci is higher than those at allozyme loci in woody plant species (*H*_*e*_ = 0.148) [51]. However, the gene diversity (expected heterozygosity under HWE) in *M*. *pauhoi* was generally comparable to those in genetically distant species assayed with SSR markers, such as 0.68 for perennial plants, 0.65 for outcrossing plants, and 0.61 for wind pollinated plants [52]. The structure of perfect flowers enables the occurrence of a mixed mating system although the level of selfing is not quantified in *M*. *pauhoi*. Most populations exhibited random mating system (insignificant *F*_*is*_), implying the pattern of predominant out crossing [17]. However, heterozygote deficit (*F*_*is*_ >>0) was present at a few loci in several populations (Table 4). As was indicated before, some of these loci with HWD were detected to arise from the occurrence of null alleles [53], such as the SHRSPa021 locus in AF, ZP, RH, YS, ZF, and PY, and the LMAV15 locus in GT, GR, and PY. A few other loci with HWD were detected to be absent of null alleles, such as LMAV15 locus in JO and GO, and the LMAV25 locus in AF, JO, and NP. Reason for these variations needs a further clarification.

The routes for population origination of *M*. *pauhoi* inferred from the genetic diversity at SSR loci are complex. Both physical (mountain ranges) and non-physical factors (Quaternary glaciations and historical human activities) could shape the pattern of genetic diversity. The physical factors include the Xuefeng, Nanling, and Wuyi Mountains, and could create barriers to inter-population gene flow. The Xuefeng Mountain (1934 m above sea level at the highest peak) exhibited a southwest-northeast orientation. The Hengduan Mountains locating at the west side of the Xuefeng Mountain also exhibit a southeast-northwest orientation. The regions between the Hengduan and Xuefeng Mountains are the high plateaus, basins, and mountainous regions (1000-2000m above sea level). This mountain shelf significantly affects the climate at the west side of the Xuefeng Mountain, and produces adverse environments that block the westward dispersal of *M*. *pauhoi*. However, the east side of the Xuefeng Mountain ranges could likely provide some refugia during the Quaternary glaciation for plants that subsequently spread southward. This possibly explains the high allele richness (*N*_*a*_) in the populations at the east side of the Xuefeng Mountain. Potential refugia or ancestral populations could likely exist around populations XA,GR, GO, GT, and ZP whose altitudes are about 52-226m above sea level (Table 1). These altitudes are much lower than the plateaus at the western side of the Xuefeng Mountain.

The Nanling Mountain ranges (1920m above sea level at the highest peak) exhibit a west-east orientation, and consist of three discontinuous and relatively small mountains. The gaps between discontinuous mountains allow the easy movement of plants from north to south or vice versa (Fig 1), forming a transitional region (aka, a biodiversity “hotspot”, [54]) where tropical and subtropical vegetation was historically mixed [55]. However, the western section harboured more deciduous broad-leaved tree species and temperate flora species than the eastern section due to the impacts of Yuunan-Guizhou Plateau (the Xuefeng Mountain is a part of the Plateau) and the flora of Central China [55]. Some relic, archaic, primitive, and endemic floras (Cathaysian flora) were maintained along the Nanling Mountain ranges [56], suggesting the existence of likely refugia for plants, insects, and animals. Historically, human admixture moving from north to south regions or from south to north regions took place along the Nanling Mountain ranges [57]. This could include the activity of seed transference, such as seedlings of *M*. *pauhoi* for the ornamental use or seeds for producing cosmetic perfume [3], [5], [6], enhancing the spread of *M*. *pauhoi*. As a consequence, barriers to gene flow were weakened among populations in this region. Genetic diversity and the number of observed alleles per locus were relatively comparable among populations (average *N*_*a*_ = 5.16 in Group II).

The Wuyi Mountain ranges (2158m above sea level at the highest peak) are continuously distributed in a southwest-to-northeast orientation. One viewpoint is that the Quaternary glaciation did not occur in the Wuyi Mountain [58] although the uncertainty about whether there was Quaternary glaciation in southern China remains to be clarified [59]. This is the same situation as in the Nanling Mountains There is no doubt that the Wuyi Mountain historically provided refugia for plants and animals [60]. A strong barrier to gene flow could exist across the Wuyi Mountain along the west-east route (Fig 1), resulting in low genetic diversity at the east side of the distribution of *M*. *pauhoi*. The eastward dispersal is weaker than the northward dispersal in the whole range-wide distribution (Table 6), and this is probably related to the strong barrier along the west-east route.

The preceding discussions suggest a general relationship between the genetic variation and the mountain barriers to gene flow in the distribution of *M*. *pauhoi*. A joint pattern for the correlations of *N*_*a*_, *H*_o_, *h*, and PIC with the geographic locations suggested a general northeastward dispersal (Table 6). As in *Ocotea ternra* [12], seed dispersal in *M*. *pauhoi* is mainly mediated by birds although dispersal through other animals cannot be excluded. Seed dispersal by water transport could not be excluded as well because many local streams run in the southwest-northeast orientation between the Xuefeng and Wuyi Mountains. This may account for the relatively close genetic relationships among populations in Group III. Pollen flow by insects could be seriously affected by the mountain barriers from the west to east sides in the species’ distribution, and hence its contribution to inter-population gene flow may be limited. As a whole, a relatively higher level of population genetic differentiation was produced in *M*. *pauhoi* than in other tropical trees [12]. The significant but weak effects of isolation-by-distance suggested that physical distance was not the main factor shaping population genetic structure. The presence of private alleles might suggest dispersal limitation. All these results supported the hypothesis that physical barriers rather than the geographic distance was the main reason that gene flow was impeded.

Populations CY, QN, LY, and YC were loosely joined with populations GO, XA, GR, and GT in Group II based on their Nei’s genetic distances (Fig 4). The speciation time for *M*. *pauhoi* in the Lauraceae family is unknown since phylogenetic relationships among species are not studied. One possible explanation is that *M*. *pauhoi* could have been widely distributed in subtropical and tropical regions in ancient periods according to the fossil records of other species in the same family [9], [10], [11], [12], [13]. Population genetic differentiation could therefore have been small in *M*. *pauhoi* before glaciations occurred in the southern regions of the Nanling and Wuyi Mountains. The subsequent glaciations, especially the Quaternary glaciation, could significantly have shaped the genetic diversity in the northern regions, but not seriously affected populations CY, QN, LY, and YC in the southern regions of the species’ distribution. In the later northeastward, or particularly the northward, dispersal [61], the previously close genetic relationships present before glaciations were maintained among the group of populations at the southeast side (CY, QN, LY, and YC) and the group of populations at the west side (XA, GO, GR, and GT) that were potentially saved in refugia. This explanation needs further evidence with additional genetic data collections.

Concerning the genetic management of *M*. *pauhoi*, a moderate level of population genetic differentiation (*G*_*st*_ = 16.91%) suggests that a strategy of considering multiple populations is needed. As discussed above, complex routes of gene flow were potentially involved in maintaining contemporary population genetic structure in *M*. *pauhoi*. Previous studies indicated that seed dispersal by birds mainly contributed to inter-population gene flow in the genus Ocotea [12], [62], [63]. This could partially occur in the genus Machilus or some other genera in the Lauraceae family [29], [64]. IBD tests indicated that the physical mountains played an important role in impeding inter-population gene flow in the distribution of *M*. *pauhoi*. Furthermore, the historical human activities in the Nanling Mountain regions could facilitate gene flow. All these processes of gene flow deserve our attention in managing natural genetic resources of *M*. *pauhoi*.

Our field survey indicated that only a small proportion of the natural distribution of *M*. *pauhoi* is present in protected nature reserves. Most populations remain in a vulnerable status and are often faced with potential deforestation. Although *M*. *pauhoi* is more widely distributed than *Persea chekiangensis* C.B. and *Persea bournei* (Hemsl.) Yang in the Lauraceae family in South China, its genetic resource is rapidly eroded due to its extensive utility for timber. Our results indicate the potential evolutionary processes forming the present population genetic structure, and this provides useful reference for genetic resource management in *M*. *pauhoi*. The three groups of populations revealed by STRUCTURE and cluster analyses (Figs 3 and 4) imply a focus on these three clusters in practical management and genetic conservation. The relatively large population genetic differentiation and Nei’s genetic distances between populations were partly attributable to high mutation rates at SSR loci, compared with those using allozyme markers [12], [19]. This might simplify practical manipulation in conserving genetic diversity because most populations in each of the three groups were loosely joined. From the distribution of genetic diversity and the northeastward dispersal, a priority should be given to the populations at the southwest sides of the distribution in conserving genetic variations (e.g., sampling for establishing a germplasm gene pool). Populations in the southwest sides could likely be more ancestral populations.

Note that the results from neutral microsatellite markers do not provide us with information on population adaptation across range-wide environmental sites [65]. SSR markers are usually considered as selectively neutral although strict tests were not conducted. Their neutrality might partially be reflected from the insignificant correlations between genetic and ecological (annual mean rainfall and annual mean temperature) components (Table 6). The neutral evolutionary processes (mutation, migration, and genetic drift) can be used to interpret the observed pattern of genetic structure. Recent provenance trials from the two-year-old seedlings showed the presence of significant differences among populations [66], implicating adaptive variation among provenances. Although results for stable growth or other adaptive phenotypic traits among provenances are not available yet, the designation of seed zones is conventionally concentrated on the basis of the provenance trials [66], such as setting up breeding populations for multiple purposes or for combating climate changes. The population structure and the historical evolutionary route inferred from SSR markers can be combined with the information from provenance trials to develop a comprehensive scheme for future genetic management [67], [68].

## Supporting information

S1 Table Diploid SSR data at twelve loci in *Machilus pauhoi*. (XLS)
